# Comparative Analysis of Clinical and MR Imaging Characteristics between Dual-Phenotype Hepatocellular Carcinoma and Conventional Hepatocellular Carcinoma: A Retrospective Study

**DOI:** 10.2174/0115734056413164251119094539

**Published:** 2025-11-29

**Authors:** Xiaoyan Yang, Caiying Wang, Mengsu Zeng, Zhengming Hu, Mingliang Wang

**Affiliations:** 1 Department of Radiology, Shenzhen Traditional Chinese Medicine Hospital, Shenzhen 518000, China; 2 Department of Pathology, Fudan University Zhongshan Hospital, Shanghai 200032, China; 3 Department of Radiology, Shanghai Geriatric Medical Center, Shanghai 200237, China; 4 Department of Ultrasound, Peking University Shenzhen Hospital, Shenzhen 518036, China; 5 Department of Radiology, Fudan University Zhongshan Hospital, Shanghai 200032, China

**Keywords:** Hepatocellular carcinoma, Liver neoplasms, Magnetic resonance imaging, Phenotype, ADC values, Lesion morphology, Cirrhosis

## Abstract

**Objective:**

This study aimed to investigate the clinical and MR imaging differences between dual-phenotype hepatocellular carcinoma (DPHCC) and conventional hepatocellular carcinoma (HCC).

**Methods:**

A retrospective analysis was conducted on the clinical data and MRI findings of 29 patients with DPHCC and 29 propensity score-matched patients with conventional HCC, confirmed by surgical pathology, from January 2019 to January 2022 at Fudan University Zhongshan Hospital. Clinical characteristics, lesion location, morphology, size, signal intensity, enhancement patterns, vascular invasion, and lymph node metastasis were analyzed for both groups.

**Results:**

Between the DPHCC group and the HCC group, statistically significant differences were found in cirrhosis, pathological grade, lesion morphology, enhancement patterns, delayed capsular enhancement, and lymph node metastasis. There were no statistically significant differences between the two groups in terms of age, gender, hepatitis B infection, AFP, CA199, microvascular invasion (MVI), capsular invasion, lesion size, location, vascular invasion, ADC values, and T1WI and T2WI signals.

**Conclusion:**

Compared to HCC, DPHCC has a higher pathological grade, more irregular lesion morphology, and a higher incidence of both fast-in and slow-out and slow-in and slow-out enhancement patterns, as well as higher rates of lymph node metastasis. The findings have provided valuable insights for the accurate diagnosis of DPHCC.

## 1 INTRODUCTION

Dual-phenotype hepatocellular carcinoma (DPHCC) is a recently identified subtype of hepatocellular carcinoma (HCC) thathasgarneredincreasingattentionin the field of hepatic oncology [1, 2]. DPHCC morphologically resembles typical HCC but is distinguished by significant immunohistochemical expression of biomarkers for both HCC and intrahepatic cholangiocarcinoma (ICC). This dual expression of biomarkers endows DPHCC with dual phenotypic characteristics, potentially manifesting the dual biological behaviors characteristic of both HCC and ICC [3].

In recent years, studies have increasingly reported on DPHCC, particularly the type expressing cytokeratin 19 (CK19), which has higher incidences of microvascular invasion, recurrence, and metastasis, and poorer clinical outcomes [4-6]. This indicates that DPHCC has a higher degree of malignancy. The definitive diagnosis of DPHCC relies on immunohistochemistry. This diagnostic method was first introduced in the 2015 edition of the Guidelines for Standardized Pathological Diagnosis of Primary Liver Cancer [7, 8].

MRI plays a crucial role in the diagnosis of HCC, with studies indicating its high sensitivity in detecting HCC and the ability to differentiate various grades of hepatocellular carcinoma with a diagnostic efficacy AUC as high as 0.841 [9]. Further studies have demonstrated that intralesional fat, tumor size, intratumoral necrosis, and other factors can predict the subtypes of HCC [10]. This integrated assessment not only heightens diagnostic accuracy, but also aids clinicians in devising more effective personalized treatment strategies. However, despite the significance of MRI in HCC diagnosis and staging, there are limited reports on the MRI findings of DPHCC.

To address this gap, the present study retrospectively analyzed 29 cases with pathologically confirmed DPHCC. We summarized their clinical data, pathological features, and MRI findings, and compared them with those of conventional HCC. The primary objectives were to determine the value of preoperative MRI in the diagnosis of DPHCC and explore its potential role in distinguishing DPHCC from conventional HCC.

## 2 MATERIALS AND METHODS

### 2.1 Study Subjects

This study was approved by the institutional review board (IRB) of Fudan University Zhongshan Hospital (approval no. B2018236) and conducted in accordance with the ethical standards of the 1964 Helsinki Declaration and its subsequent amendments. Informed consent was waived by the IRB for the use of de-identified retrospective data. The flow diagram is shown in Fig. (**1**).

#### 2.1.1 Inclusion criteria

The inclusion criteria involved patients who have undergone surgical resection and pathologically confirmed as having dual-phenotype hepatocellular carcinoma (DPHCC) or hepatocellular carcinoma (HCC) at Fudan University Zhongshan Hospital between January 2019 and January 2022; patients who have been subjected to MR plain scan and enhanced examination within two weeks prior to surgery; and patients with multiple lesions (the largest lesion was selected as the target for observation).

#### 2.1.2 Exclusion Criteria

The exclusion criteria were patients who have received treatments, such as trans-arterial chemoembolization or radiofrequency ablation, before tumor resection, and patients with incomplete clinical or imaging data.

A total of 29 DPHCC patients were enrolled, including 22 males and 7 females, aged 27 to 69 years, with an average age of 51.31 ± 11.88 years. Among the 29 patients, 16 were found to have hepatic space-occupying lesions during physical examination, 7 were identified during follow-up after HCC surgery, 3 presented with abdominal pain, 1 with diarrhea and fever, 1 with aversion to oily food, and 1 with jaundice. Of these patients, 26 had a history of chronic hepatitis B, 9 had liver cirrhosis, 21 had elevated AFP levels, and 5 had elevated CA199 levels. All 29 patients were pathologically confirmed via surgical resection.

For comparison, 29 HCC patients who met the inclusion criteria were matched using propensity score matching based on factors, such as gender and age. This group included 23 males and 6 females, aged 27 to 76 years, with an average age of 55.63 ± 11.87 years. In this group, 22 patients were found to have hepatic space-occupying lesions during physical examination, 4 presented with abdominal pain or distention, and 3 were diagnosed due to elevated AFP levels during follow-up for chronic hepatitis B. Among these patients, 28 had a history of chronic hepatitis B, 21 had liver cirrhosis, 15 had elevated AFP levels, and 3 had elevated CA199 levels. All 29 patients were pathologically confirmed via surgical resection.

### 2.2 Pathological Diagnostic Criteria

The pathological diagnosis of DPHCC and HCC was based on the 2019 WHO classification criteria for tumors of the digestive system [11]. The criteria included the following parameters: (1) the tumor consists solely of HCC components; (2) more than 15% of tumor cells exhibit strong positivity for at least one hepatocyte marker. Histological morphology and immunohistochemical staining were utilized for diagnosis. The Edmondson-Steiner grading system (ES, I-IV) was used to assess the degree of cellular differentiation.

### 2.3 MRI Equipment and Examination Methods

Magnetic resonance imaging (MRI) was performed using 3.0-T systems (United Imaging Healthcare MR 770 and Siemens Verio). The MRI protocols included unenhanced images and dynamic sequences following intravenous contrast agent injection [12]. These protocols comprised axial fat saturation (fs) T2-weighted imaging (T2WI), in-phase and out-of-phase T1-weighted imaging (T1WI), unenhanced axial fs T1WI, and dynamic triple-phase contrast-enhanced MRI (CE-MRI). Diffusion-weighted imaging (DWI) was performed using a breath-hold single-shot echo-planar imaging pulse sequence with b-values of 0 and 500 mm^2^/s, respectively. All patients received 0.2 mmol/kg body weight of gadopentetate dimeglumine (Gd-DTPA, Magnevist, Bayer Schering Pharma, Berlin, Germany) via a power injector (Spectris Solaris® EP MR, MEDRAD Inc., Indianola, IA, USA) at an infusion rate of 1.5-2 ml/s. After intravenous contrast agent injection, a three-dimensional fs T1-weighted gradient-echo sequence was used to acquire dynamically enhanced images. The arterial phase, portal phase, and delayed phase images were acquired during suspended respiration at 25-30 seconds (during monitoring, the scan is triggered when the contrast agent reaches the ascending aorta), 60-80 seconds, and 150-180 seconds, respectively. The detailed parameters of the MRI sequences are reported in Table **1**.

### 2.4 Image Analysis

MR images were independently reviewed by two experienced radiologists with associate chief physician titles or higher. The reviewers assessed the plain and multiphase enhanced MR images, focusing on lesion location (left or right lobe; for lesions at the junction of both lobes, the primary location was considered), morphology (round or irregular), size (maximum diameter), signal characteristics (compared to adjacent liver tissue), enhancement patterns (rapid wash-in and wash-out, persistent enhancement, centripetal enhancement, delayed enhancement), vascular invasion (presence of tumor thrombus), and lymph node involvement (enlarged if the short axis was ≥5 mm). ADC values of the lesions were measured three times, and the average value was taken. Discrepancies in diagnostic opinions were resolved through consultation to reach a consensus.

### 2.5 Statistical Analysis

Statistical analysis was conducted using SPSS 24.0 software. Quantitative data have been expressed as mean ± standard deviation (±s). Independent sample t-tests were used to compare quantitative data between the two types of liver cancer. Categorical data were analyzed using continuity correction chi-square tests and chi-square segmentation methods. A P-value < 0.05 was considered statistically significant.

## 3 RESULTS

### 3.1 Clinical Characteristics and Pathological Data

The clinical characteristics Table **2** and pathological data Table **3** of the two groups were compared. No statistically significant differences were observed between the DPHCC and HCC groups in terms of age, gender, hepatitis B infection, AFP, CA199, microvascular invasion (MVI), or capsular invasion. However, significant differences were found in the incidence of cirrhosis and pathological grading. Specifically, the DPHCC group had a higher incidence of cirrhosis compared to the HCC group (*P* = 0.001). Additionally, the pathological grading was more advanced in the DPHCC group, with a higher proportion of grade III tumors (*P* = 0.000). Four cases in the DPHCC group had simultaneous elevations of AFP and CA199.

### 3.2 MR Imaging Features Analysis

The analysis of MR imaging features Tables **4** and **5** showed no significant differences in lesion size, location, vascular invasion, ADC values, or T1WI and T2WI signal characteristics between the two groups. However, significant differences were found in lesion morphology, enhancement patterns, delayed capsular enhancement, and lymph node metastasis.

Among the 29 DPHCC cases, the maximum diameter ranged from 8 mm to 113 mm, with an average of 45.44 ± 32.78 mm. T1WI showed low or slightly low signal in 25 cases, with 4 cases exhibiting heterogeneous signals (2 with lower signal areas, 2 with hemorrhagic high signal areas). T2WI showed slightly high or high signals, with 13 cases exhibiting heterogeneous signals and areas of cystic necrosis. Enhancement patterns were diverse: 16 cases showed rapid wash-in and wash-out enhancement (12 non-circular, 4 circular), 9 cases showed rapid wash-in and slow wash-out enhancement (5 with centripetal filling, 4 with uniform persistent enhancement), and 4 cases showed circular slow wash-in and slow wash-out enhancement. All 29 DPHCC cases exhibited high signal on DWI and low signal on ADC. The ADC values for 27 lesions (2 lesions were affected by cardiac motion artifacts) ranged from 0.551 to 1.459 × 10^–3^ mm^2^/s, with an average of 994.69 ± 222.01 × 10^–3^ mm^2^/s. Delayed capsular enhancement was observed in 10 cases, vascular invasion in 5 cases, and lymph node metastasis in 14 cases, with the largest lymph node measuring approximately 26 mm. The MRI appearance of DPHCC is shown in Fig. (**2**).

Among the 29 HCC cases, the maximum diameter ranged from 15 mm to 138 mm, with an average of 53.75 ± 34.77 mm. T1WI showed low signal in 23 cases, with 6 cases exhibiting mixed signals due to hemorrhage. T2WI showed slightly high signal in 15 cases, with heterogeneous signals and areas of cystic high signal. Enhancement patterns included 28 cases with rapid wash-in and wash-out enhancement (25 non-circular, 3 circular) and 1 case with rapid wash-in and slow wash-out enhancement. All 29 HCC cases exhibited high signal on DWI and low signal on ADC. The ADC values for 26 lesions (3 lesions were affected by artifacts) ranged from 0.425 to 1.535 × 10–3 mm^2^/s, with an average of 1.002 ± 0.229 × 10–3 mm^2^/s. Delayed capsular enhancement was observed in 20 cases, vascular invasion in 2 cases, and lymph node metastasis in 1 case, with the largest lymph node measuring approximately 8 mm. The MRI appearance of HCC is shown in Fig. (**3**).

## 4 DISCUSSION

Primary liver cancer stands as one of the most prevalent malignant tumors globally [13, 14]. According to the 2022 China Cancer Statistics Report, primary liver cancer ranks 5th in terms of incidence and 2nd in mortality among all malignancies, with HCC accounting for 75-85% of cases [15]. Different subtypes of HCC exhibit various genetic mutations and molecular profiles, which translate to remarkable differences in patient prognosis [16]. Dual-phenotype hepatocellular carcinoma (DPHCC) is a unique subtype of HCC, distinguished by specific immunophenotypic features, distinct molecular mechanisms, and notable clinical outcomes. DPHCC is characterized by a high postoperative recurrence rate and low patient survival rate, comprising approximately 10% of HCC cases [17, 18].

### 4.1 Pathological Diagnosis Criteria of DPHCC [7, 8]

The pathological criteria for the diagnosis of DPHCC are as follows: (1) the tumor consists solely of HCC components; (2) more than 15% of tumor cells exhibit strong positivity for at least one hepatocyte marker (*e.g*., AFP, Hep Par-1, GPC-3, CK8) and at least one cholangiocyte marker (*e.g*., CK7, CK19).

Hepatocytes, bile duct epithelial cells, and hepatocarcinoma cells all contain cytoskeletal intermediate filaments. Different types of cancer cells exhibit distinct characteristic combinations of cytokeratins (CK). Hepatocytes and conventional hepatocellular carcinoma (HCC) typically express CK8 and CK18, while cholangiocytes and cholangiocarcinoma (ICC) generally express CK7 and CK19. Although CK7 and CK19 are both expressed in cholangiocytes, CK7 is also recognized as a marker of intermediate hepatocytes, and CK19 serves as a marker of ICC and biliary differentiation [19].

This is different from classical combined hepatocellular-cholangiocarcinoma (CHC), where a single tumor nodule contains distinct HCC and intrahepatic cholangiocarcinoma (ICC) components, each expressing their respective immuno-histochemical markers. Therefore, pathologically, DPHCC presents typical HCC histological and cytological features, but differs from ordinary HCC in that its immunohis-tochemical staining shows expression of both HCC and ICC biomarkers. Diagnosis relies mainly on immunohistochemical staining.

### 4.2 Pathogenesis

Compared to cholangiocyte markers, like CK7 and MUC-1, CK19 shows higher sensitivity and specificity, making it one of the most commonly used markers for the pathological diagnosis of cholangiocarcinoma and DPHCC [20]. CK19 can regulate the malignant biological behavior of DPHCC through multiple pathways and is also an important indicator of poor prognosis. Feng *et al*. found CK19-positive HCC to exhibit more aggressive biological behavior than conventional HCC [21].

### 4.3 Clinical Characteristics and Pathological Data

In this study, DPHCC predominantly affected middle-aged to elderly males, most of whom had a history of chronic hepatitis B and elevated serum AFP levels, showing no significant differences from HCC. These findings are consistent with domestic and international reports. Some studies suggest that DPHCC is more prone to microvascular invasion (MVI) and capsular invasion compared to HCC. Studies by Feng and Wang found higher incidences of MVI (62.3% vs. 41.2%) and capsular invasion (61.3% vs. 34.0%) in DPHCC [22, 23]. However, no significant differences were observed in our study (48.3% vs. 37.9%, 51.7% vs. 62.1%), possibly due to the study being conducted at a regional medical center, where patients tend to have more severe conditions. A small portion of DPHCC patients (17.2%) showed elevated CA199 levels, with 4 cases (13.8%) having simultaneous elevations of AFP and CA199, similar to HCC. Although tumor cells in DPHCC can produce both AFP and CA199, and simultaneous elevations of these markers in serum may suggest DPHCC, only a minority of DPHCC patients exhibit this clinical feature.

Cirrhosis was more common in the HCC group. HCC often develops on a background of hepatitis and cirrhosis, with regenerative nodules progressing to dysplastic nodules and eventually to HCC [24, 25]. In contrast, DPHCC, with its dual biological characteristics of HCC and ICC, is more aggressive and progresses rapidly, often without a significant cirrhosis background [26, 27]. This biological characteristic also explains the poorer differentiation of tumor cells in DPHCC and the differences in pathological grading between the two groups, with more cases of grade III in the DPHCC group (86.2%) and more cases of grade II in the HCC group (69.0%).

### 4.4 MRI Features

No significant differences were found between the two groups in T1WI and T2WI signals. However, DPHCC more frequently exhibited fast-in and slow-out or slow-in and slow-out enhancement patterns (13/29, 44.8%), similar to ICC [28, 29]. The DPHCC group also had more irregular lesion shapes and a higher incidence of abdominal and/or retroperitoneal lymph node metastasis compared to the HCC group. Hepatic progenitor cells, which possess bidirectional differentiation potential, play a role in liver repair following injury. Some researchers believe that DPHCC may originate from hepatic progenitor cells, maintaining their bidirectional differentiation potential and exhibiting dual biological behaviors. CK7 and CK19, as endogenous components of biliary epithelial cells, are overexpressed in hepatocytes, leading to MRI findings that resemble those of ICC.

The results of this study indicated that, compared to conventional HCC, DPHCC less frequently exhibits fast-in and fast-out enhancement, as well as delayed capsule enhancement. The differences between the two are statistically significant.

These MRI features may be related to the dual biological behavior of tumor cells in DPHCC, which have stronger proliferative and invasive capabilities [30]. Some lesions resemble HCC and show fast-in and rapid-out enhancement, primarily supplied by the hepatic artery [31]. Other lesions exhibit centripetal filling and delayed enhancement, possibly indicating a dual blood supply from both the hepatic artery and portal vein, or a tumor structure similar to ICC with corresponding enhancement characteristics [32].

Although the current treatment regimens for both DPHCC and conventional HCC are mainly surgery-based, the differences in imaging features identified in this study (such as the rim enhancement pattern and a higher tendency for lymph node metastasis) can provide important references for clinical diagnosis and treatment. For instance, these features are conducive to more accurate preoperative prediction of tumor biological behaviors, thereby assisting in optimizing the formulation of surgical plans and postoperative follow-up strategies.

## 5 LIMITATIONS

Our study involved several limitations. First, this study only included surgical patients, potentially introducing selection bias. Additionally, it did not include liver cancer caused by hepatitis C or alcoholic hepatitis. Second, this study was retrospective and had a relatively small sample size, which can be attributed to the low incidence of DPHCC. Future studies on DPHCC should enroll non-surgical patients, involve multicenter, large-sample prospective research studies (to reduce selection bias),and incorporate new MRI technologies and methods in order to provide more comprehensive insights into its characteristics and improve diagnostic and treatment strategies.

## CONCLUSION

Compared to HCC, DPHCC has been found to have a higher pathological grading level, irregular lesion morphology, fast-in and slow-out or slow-in and slow-out enhancement patterns, and a higher incidence of lymph node metastasis. The incidence of cirrhosis and delayed capsular enhancement was lower. This information offers valuable guidance for the precise diagnosis of DPHCC, prompting clinicians to adopt more aggressive treatment strategies.

## Figures and Tables

**Fig. (1):**
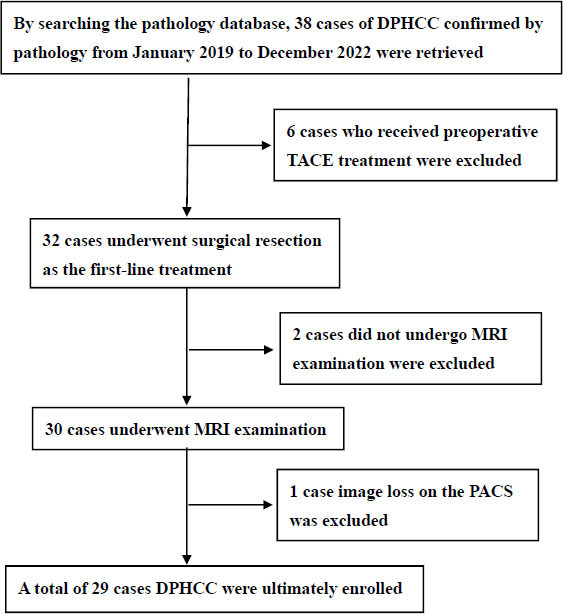
Flow diagram.

**Fig. (2):**
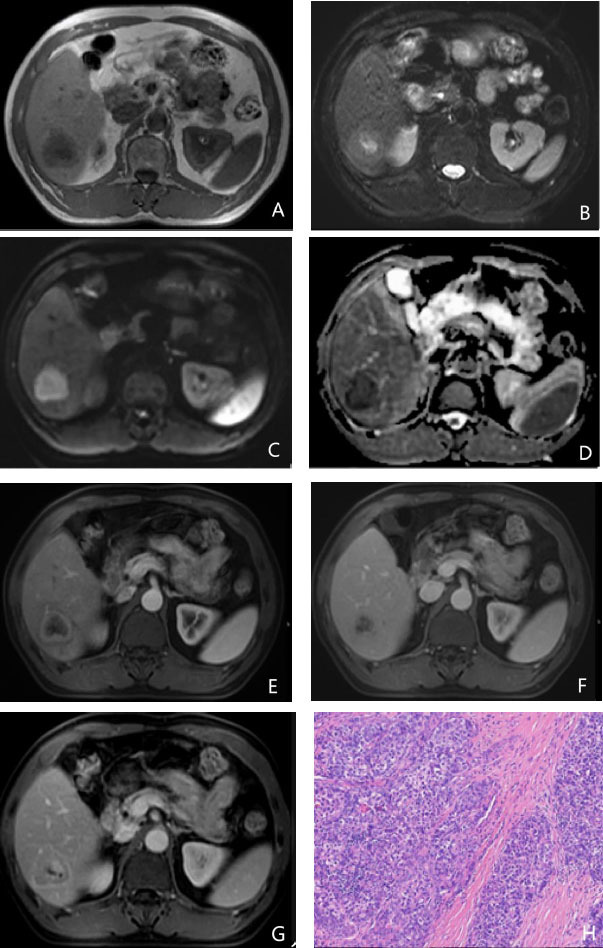
A 59-year-old male DPHCC patient. **A**: T1WI showed low signal. **B**: T2WI showed a heterogeneous, slightly high signal. **C**: DWI showed a high signal. **D**: **ADC** showed a low signal. **E**, **F**, **G**: After enhancement, the lesion showed ring-like high enhancement in the arterial phase and progressive enhancement in the portal venous and delayed phases. **H**: Pathological observation (10×) showed many fibrous tissues in the tumor.

**Fig. (3):**
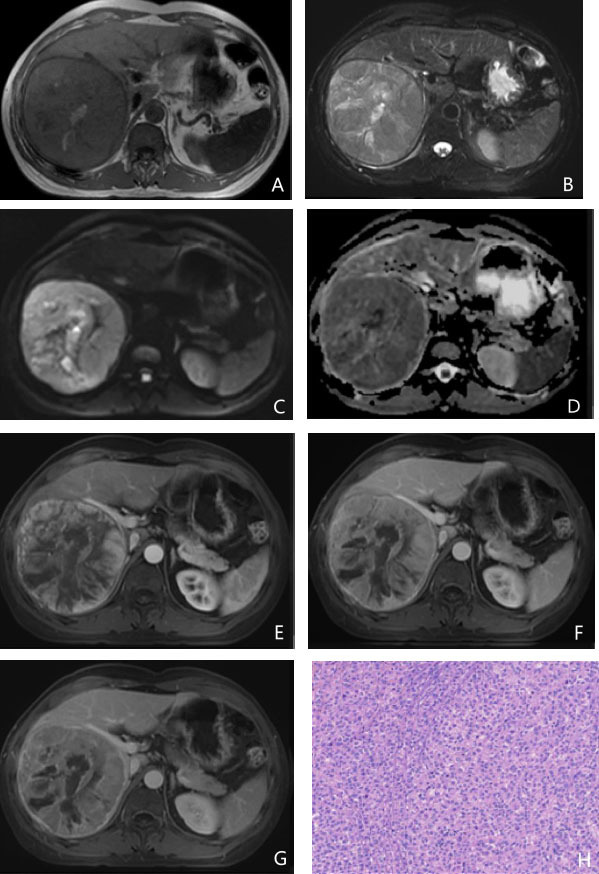
**A** 49-year-old male HCC patient. A: T1WI showed low signal as well as high signal in the center. **B**: T2WI showed a heterogeneous high signal. **C**: DWI showed a high signal. **D**: ADC showed a low signal. **E**, **F**, **G**: After enhancement, the lesion showed diffuse heterogeneously high enhancement in the arterial phase and decreased enhancement in the portal venous and delayed phases, with capsular enhancement. **H**: Pathological observation (10×) showed that tumor nuclei were large and had less surrounding fibrous tissues.

**Table 1:** MR imaging sequences and parameters.

**Parameter**	**T2WI-FS**	**T1W IP-OP**	**T1W-FS tra**	**T1W-FS cor**	**DWI**
**3.0-T UIH MR 770**					
Repetition time (msec)	2000	4.27	3.3	3.3	4165
Echo time (msec)	106.2	2.5 and 1.21	1.5	1.5	66.3
Matrix size	256×256	288×168	320×216	288×208	128×101
Field of view (mm^2^)	346×346	300×300	270×270	340×340	300×300
Slice thickness (mm)	6–7	3–4	3	3–4	6–7
Slice gap (mm)	1.8–2.1	0	0	0	1.8–2.1
Average	1	1	1	1	5
**Verio 3.0-T Siemens**					
Repetition time (msec)	2000–3000	207	4.17	4.07	3400
Echo time (msec)	83	2.31 and 3.69	1.43	1.46	70
Matrix size	320×165	256×141	352×200	384×269	128×80
Field of view (mm^2^)	285×380	285×380	285×380	380×380	285×380
Slice thickness (mm)	5.5	5.5	3	3	6
Slice gap (mm)	1.1	1.1	0	0	1.8
Average	1	1	1	1	4

**Table 2:** Comparison of clinical data of two types of hepatocellular carcinomas.

Variables	Age (years)	Gender		HBV Infection		Hepatocirrhosis		AFP		CA199	
		Male	Female	Yes	No	Yes	No	Positive	Negative	Positive	Negative
DPHCC (N=29)	51.31±11.88	22	7	26	3	9	20	21	8	5	24
HCC (N=29)	55.48±12.05	23	6	28	1	20	9	15	14	3	26
Statistical value	-1.327 ^a^	0.099^b^		1.074^b^		11.582^b^		2.636^b^		0.580^b^	
*p*-value	0.190	0.753		0.300		0.001		0.104		0.446	

**Note:**a:*t* value; b:*χ2* value.

**Table 3:** Comparison of pathological data of two types of hepatocellular carcinomas.

Variables	Pathological Grading			MVI Grading			Capsule Invasion	
	I	II	III	0	1	2	Yes	No
DPHCC (N=29)	0	4	25	15	8	6	15	14
HCC (N=29)	0	20	9	18	10	1	18	11
*χ2* value	18.196			0.406			0.633	
*p*-value	0.000			0.131			0.426	

**Note:** The chi-square partitioning method was utilized for the statistical analysis of the differences between pathological grading and MVI grading.

**Table 4:** Comparison of morphological signs of two types of hepatocellular carcinomas.

Variables	Morphology		Maximum Diameter (mm)	Location		Vascular Invasion		Lymph Node Metastasis		ADC Value
	Regular	Irregular		Right lobe	Left lobe	Yes	No	Yes	No	
DPHCC (N=29)	16	13	45.44±32.77	23	6	5	24	14	15	994.69±222.01 1022.24±231.35
HCC (N=29)	25	4	53.75±34.77	20	9	2	27	1	28	
Statistical value	6.740^a^		-0.933^b^	0.809^a^		1.462^a^		15.197^a^		0.055^b^
*p*-value	0.009		0.857	0.368		0.227		0.000		0.815

**Note:** a:*χ2* value; b:*t* value.

**Table 5:** Comparison of signals and signs of enhancement between the two types of hepatocellular carcinomas.

Variables	T1WI		T2WI		Arterial Phase Enhancement		Contrast-enhanced Pattern			Delayed Capsular Enhancement	
	Low Intensity	Heterogeneous Intensity	Homogeneous	Heterogeneous	Rim	Not Rim	Fast-in and Fast-out	Fast-in and Slow-out	Slow-in and Slow-out	Yes	No
DPHCC (N=29)	25	4	16	13	8	21	16	9	4	10	19
HCC (N=29)	23	6	15	14	3	26	28	1	0	20	9
χ2 value	0.483		0.069		2.805		10.712			5.145	
*p*-value	0.487		0.792		0.094		0.005			0.023	

**Note:** The chi-square partitioning method was utilized for the statistical analysis of the differences between contrast-enhanced patterns.

## Data Availability

The authors confirm that the data supporting the findings of this research are available within the article.

## LIST OF ABBREVIATIONS

DPHCC: Dual-phenotype hepatocellular carcinoma
HCC: Hepatocellular carcinoma
ICC: Intrahepatic cholangiocarcinoma
MVI: Microvascular invasion
MRI: Magnetic resonance imaging
ADC: Apparent diffusion coefficient
DWI: Diffusion-weighted imaging
T1WI: T1-weighted imaging
T2WI: T2-weighted imaging

## STANDARDS OF REPORTING

STROBE guidelines were followed.
